# Drought Atlas of India, 1901–2020

**DOI:** 10.1038/s41597-023-02856-y

**Published:** 2024-01-02

**Authors:** Dipesh Singh Chuphal, Anuj Prakash Kushwaha, Saran Aadhar, Vimal Mishra

**Affiliations:** 1 Civil Engineering, Indian Institute of Technology (IIT) Gandhinagar, Gandhinagar, India; 2 Earth Sciences, Indian Institute of Technology (IIT) Gandhinagar, Gandhinagar, India; 3 Civil & Infrastructure Engineering, Indian Institute of Technology (IIT) Jodhpur, Jodhpur, India

**Keywords:** Hydrology, Natural hazards

## Abstract

India has been considerably affected by droughts in the recent past. Despite the considerable impacts of droughts on agriculture and water resources, long-term datasets to examine droughts and their consequences at appropriate spatial and temporal scales have been lacking in India. Datasets that provide drought information are mostly available for a short period and at coarser resolutions, therefore, these do not comprehend the information regarding the major droughts that occurred in the distant past at administrative scales of decision-making. To fill this critical gap, we developed the high-resolution (0.05°) and long-term monthly precipitation and temperature datasets for the 1901–2021 period. We used long-term high-resolution precipitation and temperature to estimate droughts using standardized precipitation and evapotranspiration index (SPEI). As SPEI considers the role of air temperature in drought estimation, it can be used to examine meteorological, agricultural, and hydrological droughts. Using high-resolution SPEI, we developed drought atlas for India (1901–2020) that can provide comprehensive information on drought occurrence, impacts, and risks in India.

## Background & Summary

Droughts are hydroclimatic extreme events that lead to prolonged periods of water scarcity, impacting agricultural production and food security worldwide^1,2^. Specifically, in monsoon-dominated regions like India, droughts have been recurrent^3–5^ and caused major famines in the 19^th^ and 20^th^ centuries^6^. The southwest monsoon rainfall in India is the primary source of agricultural water^7^ and groundwater recharge^8,9^, accounting for 80% of the total annual rainfall. Droughts in India due to the weakening of the southwest monsoon are closely linked to Indian Ocean warming and El Nino/Southern Oscillation (ENSO)^7,10–12^. Also, the diverse physiographic conditions and significant variability in rainfall patterns across India contribute to the varying intensities of drought events^13^.

India is highly vulnerable to drought with about two-thirds of its area prone to drought^14–16^. Being an agricultural-dominant country and home to 1.4 billion people, droughts in India profoundly impact agricultural productivity, water resource management, and socio-economic well-being. India has witnessed a rise in the frequency, severity, and duration of droughts over the recent decades, which is projected to be further exacerbated by climate change^4,10,17–19^. With the increasing food demand due to rising population and urbanization^20,21^, the impact of droughts is expected to become more severe in the future. Additionally, unsustainable pumping of groundwater adds further to the drought-induced challenges, increasing the risks in the future^22,23^.

Understanding the observed droughts and their patterns is crucial to reduce the vulnerability of India’s population to future drought events. Trends and variability of droughts in the Indian monsoon region have been greatly examined, however, mostly at a coarser spatial resolution^3,10^. Additionally, there have been studies on a particular region^24,25^ and for a specific drought year^17,26^. While Aadhar & Mishra^27^ developed high-resolution precipitation and temperature for monitoring droughts in South Asia, its temporal coverage is limited from 1981 to 2020. Therefore, the available high-resolution datasets do not provide information on the severe droughts that occurred in the distant past. Despite its importance for the climate change adaptation and decision making, the long-term (1901–2021) high-resolution (0.05°) drought product for India has been lacking. Long-term reconstruction of droughts at higher spatial resolution is crucial to understand the impacts of some of the worst droughts in the past at local and regional scales. In addition, high-resolution and long-term drought reconstruction can be valuable for climate change adaptation, providing insights for policy interventions. Most of the available drought-related data sets are at coarser spatial resolution or with limited temporal coverage. To fill these crucial research gaps that hinder the decision-making at a local scale (Taluk level), we develop a high-resolution and long-term gridded drought assessment product based on the Standardized Precipitation Evapotranspiration Index (SPEI)^28,29^ spanning the period from 1901–2021. We developed the high-resolution and long-term monthly precipitation and air temperature datasets for the 1901–2021 period to estimate the SPEI, which overcomes the limitations of the Palmer Drought Severity Index (PDSI)^30^ and Standardized Precipitation Index (SPI)^31^ by taking into account the multi-scale characteristics of droughts and the influence of rising temperatures on atmospheric water demand. The high-resolution SPEI dataset is then used to develop a long-term drought atlas^32–34^ of India, which can assist in policymaking, disaster-risk management, and climate change adaptation.

## Methods

### Workflow

**Fig. 1:**
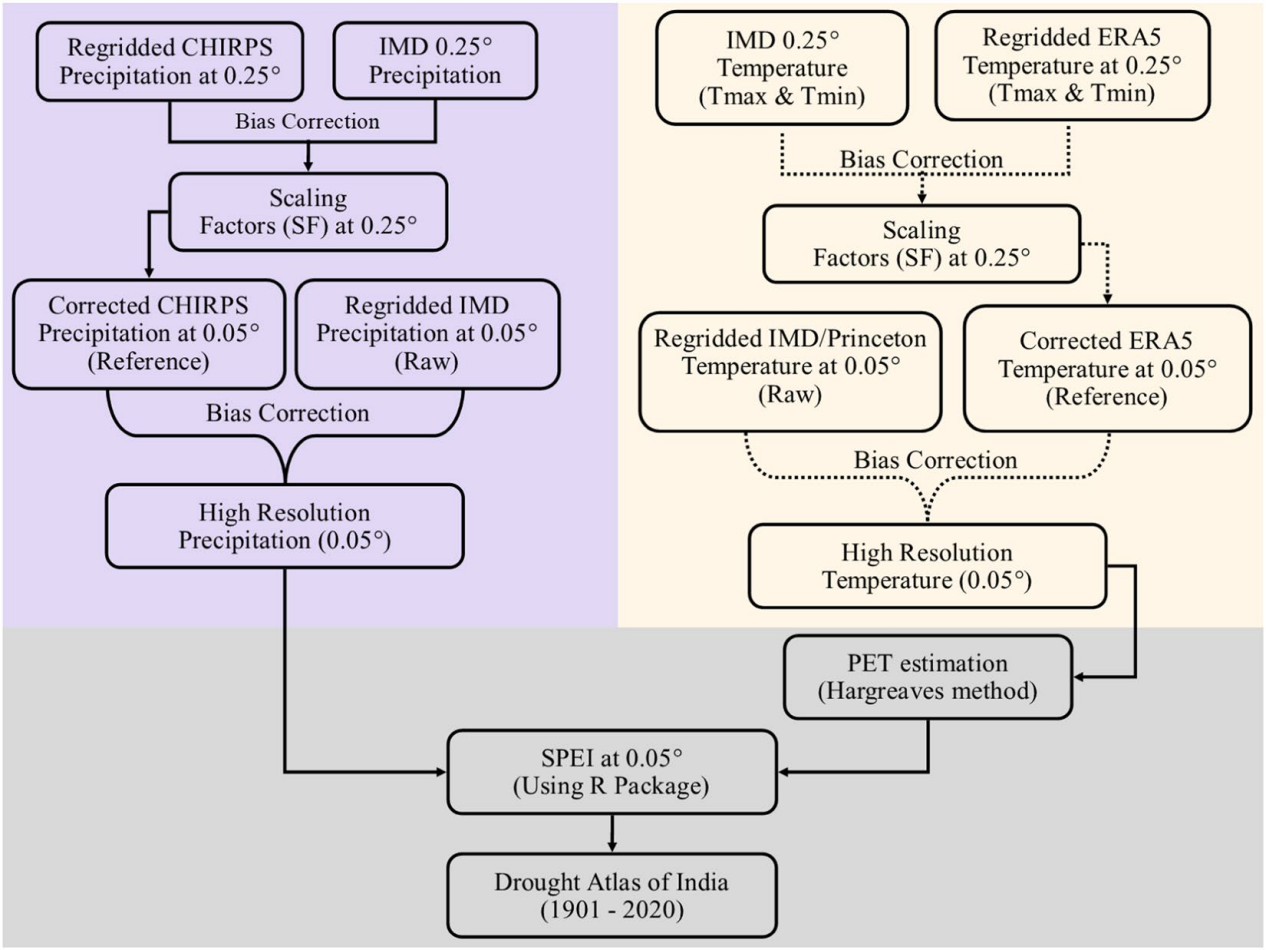
Flow chart of the overall methodology used to develop drought atlas for India.

We developed a drought atlas of India using high-resolution (0.05°) precipitation and maximum and minimum temperatures. The existing observed precipitation and temperature for India are available at the coarser spatial resolution (0.25°) for the 1901–2021 period. We developed high-resolution gridded precipitation and temperature by integrating the high-resolution products available for shorter periods and using the Quantile-Quantile (QQ) mapping for bias-correction. The bias-corrected precipitation from CHIRPS^35^ at 0.05° was used as the reference data for correcting the gridded precipitation from IMD^36^ at 0.05°. Similarly, bias-corrected temperature from ERA5-Land reanalysis^37^ was used to correct gridded temperature^38,39^ at 0.05°. The performance of high-resolution data in terms of bias, seasonality, and spatial pattern was carefully examined against the bias-corrected CHIRPS precipitation and ERA5-Land temperature. The flow chart of the overall methodology to develop the drought atlas of India is shown in Fig. 1.

### Development of high-resolution precipitation and air temperature dataset

We used satellite-based and reanalysis data products from CHIRPS and ERA5-Land to develop high-resolution precipitation and temperature. However, these hybrid datasets (CHIRPS and ERA5-Land) exhibit bias in space and time compared to observed datasets due to inadequate sampling, lack of ground-based observations, and error correction processes^40,41^. Consequently, the direct application of these datasets in studies related to climate change and hydroclimatic extremes may not be appropriate and straightforward. Several bias correction methods have been developed to address this challenge^42–47^. Bias correction involves a statistical transformation to modify the distribution of modelled data so that it closely resembles the observed data. We used the distribution (Quantile-Quantile) mapping bias correction method to reduce the bias in these datasets and making them consistent with the observed datasets. The distribution mapping method efficiently reduces bias for mean and interannual variations and also for extreme events^48^. Aadhar & Mishra^27^ compared linear scaling^27,49,50^ and distribution mapping^43,50^ for the bias correction of precipitation and temperature over South Asia and demonstrated that distribution mapping performs better than the linear scaling. Detailed information on distribution mapping methods is available in previous studies^27,43,49^.

**Fig. 2:**
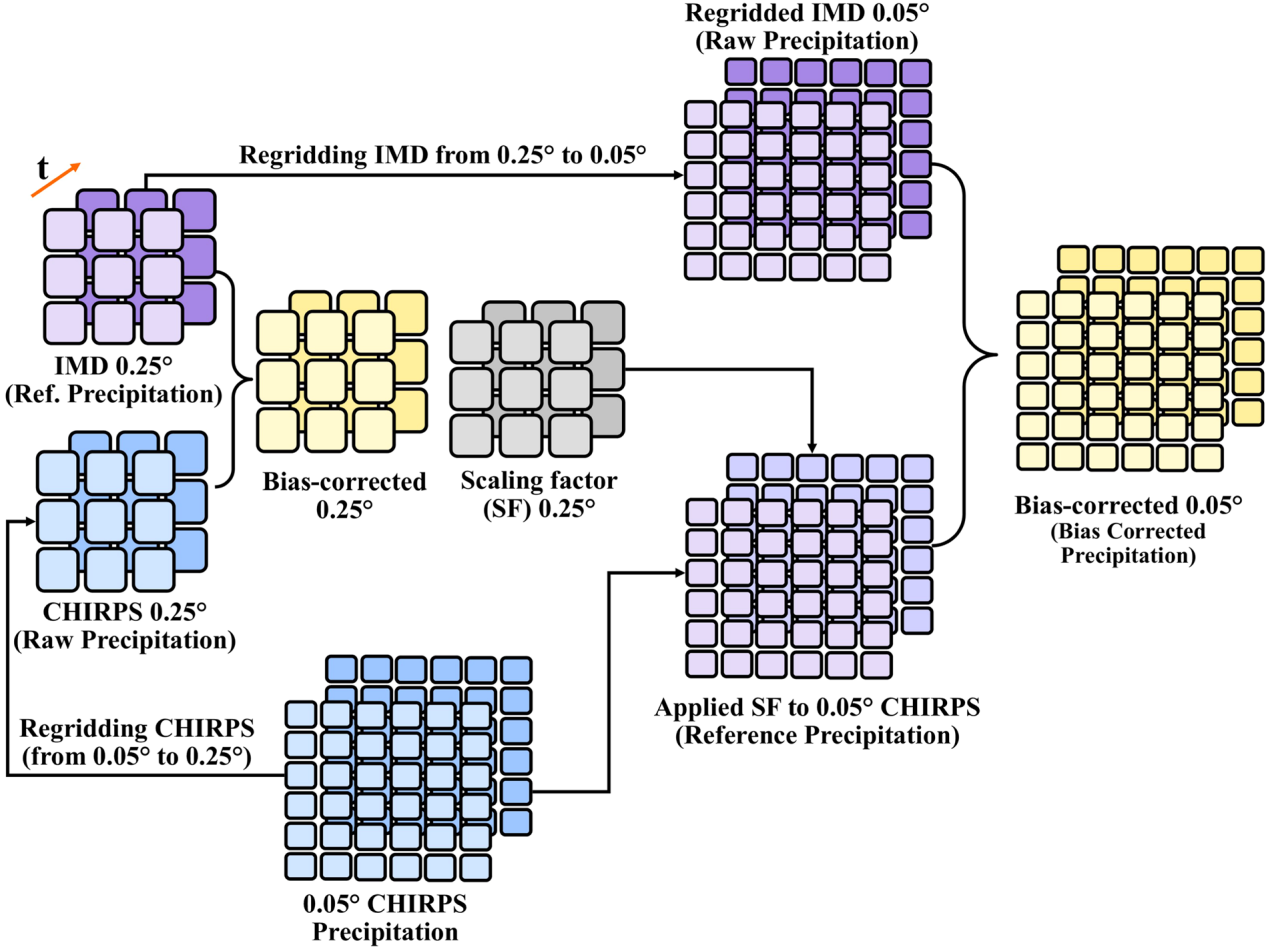
Steps to construct high-resolution (0.05°) precipitation data.

The high-resolution bias-corrected gridded precipitation was developed using gridded precipitation from India Meteorological Department (IMD) and CHIRPS. IMD precipitation is available for 1901–2021 at 0.25° spatial resolution, while CHIRPS precipitation is available from 1981 to 2021 at 0.05° spatial resolution. Since CHIRPS precipitation is a combined product of satellite observations, *in-situ* data, and observed climatology^35,51^, it has bias and random errors^27,52^. To remove the bias, first, we aggregated the CHIRPS precipitation from 0.05° to 0.25° spatial resolution to perform the bias correction. Next, we bias-corrected the aggregated CHIRPS precipitation (Raw data) using the IMD precipitation (Reference data) at 0.25° spatial resolution for the period 1981–2021. The bias correction of CHIRPS precipitation was performed using the distribution (Quantile-Quantile) mapping method as described in Aadhar & Mishra^27^. During the bias correction of CHIRPS precipitation at 0.25°, scaling factors (SF) were estimated for the distribution mapping. Further, these scaling factors estimated at 0.25° were also applied to bias-correct the CHIRPS precipitation data at 0.05° spatial resolution. Considering the bias-corrected CHIRPS precipitation at 0.05° as reference data, we bias-corrected the regridded IMD precipitation (Raw data) at 0.05° to construct the high-resolution and long-term precipitation data over India. The bias correction of IMD precipitation at 0.05° was performed using the same distribution mapping method. The stepwise description to construct the bias-corrected high-resolution precipitation data from 1901 to 2021 is shown in Fig. 2. The overall methodology to develop high-resolution precipitation product is described in detail in Aadhar and Mishra^27^.

Next, we constructed the high-resolution and long-term maximum and minimum air temperatures over India using gridded temperatures from IMD, Princeton^38^, and ERA5-Land reanalysis. Maximum and minimum temperature from IMD is available for 1951–2021 at the spatial resolution of 0.25°. Gridded temperature from IMD is unavailable for the 1901–1950 period, therefore, we used the bias-corrected temperature from the Princeton database for the 1901–1950 period at 0.25° spatial resolution. The Princeton temperature data has been used in several hydrological applications in the Indian subcontinent^18,53,54^. The bias correction of Princeton temperature was performed using the same distribution mapping method^43,50^. The temperature data from Princeton was bias-corrected against IMD for the period 1951–2010 and scaling factors were estimated. The scaling factors were applied to bias-correct the Princeton temperature for the period 1901–1950 at the spatial resolution of 0.25°. Finally, the bias-corrected Princeton temperature for the 1901–1950 period and IMD temperature for the 1951–2021 period at 0.25° spatial resolution were used for further analysis.

To construct the high-resolution temperature data, we used the ERA5-Land temperature for the period 1951–2021 at 0.1° spatial resolution. ERA5-Land reanalysis is also a combined product of weather models and observations from the satellite and *in-situ* measurements^37^. Compared to the observed datasets, ERA5-Land reanalysis consists of bias in air temperature^48^. Therefore, the bias correction of ERA5-Land temperature (Raw data) was performed using the observed IMD temperature data (Reference data) at the spatial resolution of 0.25°. To perform the bias correction, the ERA5-Land temperature was aggregated from 0.1° to 0.25° spatial resolution. The correction in aggregated ERA5-Land temperature was performed using the distribution mapping and scaling factors were estimated at spatial resolution of 0.25°. Similar to precipitation, the scaling factors were applied to bias-correct the ERA5-Land temperature at 0.05°. We constructed the high-resolution ERA5-Land temperature at 0.05° from 0.1° spatial resolution using the elevation-based SYMAP algorithm^55–57^. The SYMAP algorithm^55^ was also used to regrid the bias-corrected Princeton and IMD temperature data (Observed-Temperature data) at 0.05° from the spatial resolution of 0.25° for the period 1901–2021. Finally, we used the bias-corrected ERA5-Land temperature (Reference data) at 0.05° to bias-correct the regridded observed temperature data (Raw data) at the spatial resolution of 0.05° for the period 1901–2021 using the distribution mapping method. The stepwise description to construct the bias-corrected high-resolution temperature data from 1901 to 2021 is shown in Figure S1.

### Development of high-resolution and long-term drought index

We estimated high-resolution and long-term (1901–1921) SPEI to analyze droughts in India. SPEI is a standardized index that depends on both precipitation and potential evapotranspiration (PET), incorporating the impact of temperature on atmospheric water demand^31^. SPEI primarily focuses on meteorological aspects and does not directly incorporate agricultural or hydrological factors, such as soil moisture or streamflow. However, SPEI at an appropriate duration can be well correlated with streamflow and soil-moisture based drought indicators. We used high-resolution bias-corrected maximum and minimum temperature data to estimate PET. We employed the Hargreaves method^58^ for estimating PET due to the inadequacy of meteorological observations required for the Penman-Monteith method^59^. We fitted the log-logistic distribution to the data and estimated the SPEI values using the available SPEI package in R^60^. We categorized the SPEI values into distinct drought categories as abnormal drought (−0.8 to −0.5), moderate drought (−1.3 to −0.8), severe drought (−1.6 to −1.3), extreme drought (−2.0 to −1.6), and exceptional drought (less than −2.0) in our study^27,61^. The SPEI values greater than −0.5 indicate normal or wet conditions. PET based on the Hargreaves method can be estimated as:

1 $$PET=0.0023\ast {R}_{A}\ast {({T}_{max}-{T}_{min})}^{0.5}\ast (T+17.8).$$

where R_A_ represents mean monthly extra-terrestrial radiation (MJm^−2^/day), which is a function of latitude and day of the year^59^, T_max_ represents monthly mean daily maximum temperature (°C), T_min_ represents monthly mean daily minimum temperature (°C), and T represents monthly mean temperature (°C).

SPEI was estimated at 1-month, 4-month, and 12-month time scales. The 1-month SPEI is essential for assessing the short-term meteorological drought and supports immediate decision-making. The 4-month SPEI monitors seasonal drought or wet conditions, providing insights into agricultural droughts. In contrast, the 12-month SPEI is more suitable for assessing the impact of droughts on surface and groundwater resources. We used 1-month SPEI to estimate monthly drought conditions for the summer monsoon months (JJAS) individually. We used 4-month SPEI at the end of September and January to estimate drought conditions for the summer monsoon and winter monsoon (ONDJ), respectively. Moreover, 12-month SPEI at the end of December and May were used to estimate drought conditions for the calendar year (Jan-Dec) and water year (Jun-May), respectively. Further, the gridded SPEI was used to evaluate the mean SPEI for India at country, states (including union territories), districts, and taluka (sub-district) levels. We computed mean SPEI for grids corresponding to each geographical level (country, states, districts, and talukas).

## Data Records

The drought atlas of India covering the period 1901–2020 at the taluka level has been made available through the Zenodo repository^62^. The repository also includes the gridded SPEI values at 1-month, 4-month, and 12-month time scales for India at 0.05° spatial resolution from 1901 to 2021. Moreover, standardized SPEI corresponding to different geographical levels has also been aggregated in the repository. Interested users can refer to the readme file available in the same repository for information regarding the data format and details.

## Technical Validation

We bias-corrected the raw CHIRPS precipitation aggregated at 0.25° against the reference IMD precipitation for the period 1981–2016 (Figure S2). The raw CHIRPS precipitation exhibited both dry and wet biases in the mean annual precipitation (Figure S2A). Raw precipitation underestimated rainfall in the Kutch region, lower Himalayas, and parts of the Western Ghats while overestimated in Northeast India and South India regions. The bias-corrected CHIRPS precipitation showed a considerably lower bias for most regions of India than the raw CHIRPS precipitation (Figure S2A,B). We compared the monthly mean climatology of raw (CHIRPS), reference (IMD), and bias-corrected (CHIRPS) precipitation (Figure S2C). The corrected precipitation showed a good agreement with reference precipitation (Figure S2C). We compared the mean annual IMD regridded precipitation (raw) and bias-corrected high-resolution precipitation (corrected) against the reference precipitation (bias-corrected CHIRPS) at 0.05° for the period 1981–2020 (Figure S3). We note that the spatial variability of the reference precipitation was well represented in the bias-corrected high-resolution precipitation (Figure S3B,C), however, we find some differences in the raw precipitation (Figure S3A). Both datasets (reference and corrected) effectively captured the regions with high (North-East India, Western Ghats) and low (parts of Rajasthan and Western India) mean annual precipitation. Furthermore, we compared the mean monthly bias-corrected high-resolution precipitation against CHIRPS (already available high-resolution precipitation) data available at 0.05°.

We find a significant difference in all-India averaged monthly rainfall from 1981 to 2020 between the two precipitation datasets (Figure S3d). We quantified the performance improvement due to bias correction of the mean monthly precipitation over India by evaluating the Nash-Sutcliffe efficiency (NSE)^63^, coefficient of determination (R^2^), and root-mean-square error (RMSE). We find an increase in NSE from 0.96 to 0.98, while the R^2^ improved from 0.97 to 0.99 after the bias correction. Moreover, the RMSE for monthly precipitation was reduced from 12 to 8 mm/month after the bias correction. Evaluation of NSE, R^2^, and RMSE for the homogenous rainfall zones (Figure S8) also showed significant improvements after the bias correction (Table S1). For instance, in the case of Hilly regions, NSE increased from 0.36 to 0.78, R^2^ increased from 0.73 to 0.79, and RMSE decreased from 57 to 33 mm/month.

Similar to precipitation, we bias-corrected the raw ERA5-Land temperatures (maximum and minimum) aggregated at 0.25° against the reference IMD temperature for 1981–2016 (Figure S4). We observed a predominantly cold bias over the Indian region in ERA5-Land maximum temperature, except for the Kutch region (Figure S4A). In contrast, the ERA5-Land minimum temperature exhibited a warm bias in most areas (Figure S4d). Nevertheless, a significant reduction in bias was observed after the bias correction (Figure S4B,E). Additionally, we compared the monthly mean climatology of raw (ERA5-Land), reference (IMD), and bias-corrected (ERA5-Land) maximum and minimum temperatures (Figure S4C,F). We find that the corrected temperatures exhibited a good agreement with the reference IMD temperature. We also bias-corrected the Princeton temperature (maximum and minimum) before 1950 against IMD-Temperature (refer to Methods for detail) at 0.25° (Figure S5). The mean annual Princeton-Temperature over India before 1950 showed a significant cold bias of 3 °C compared to IMD-Temperature after 1950 (Figure S5A,C). Nonetheless, a consistent temperature trend was observed between 1901–2010 after the bias correction (Figure S5B,D). The bias-corrected Princeton temperature (1901–1950) and IMD temperature (1951–2021) were regridded at 0.05° spatial resolution, which were used as raw data to construct the long-term high-resolution temperature data.

Next, we compared the mean annual regridded IMD (raw) temperatures (maximum and minimum) and bias-corrected high-resolution temperatures against the reference temperatures (bias-corrected ERA5-Land) at 0.05° for the period 1981–2016 (Figures S6, S7). The spatial variability of the reference temperature was well represented in the bias-corrected high-resolution maximum (Figure S6B,C) and minimum (Figure S7B,C) temperature. The significant difference in monthly mean ERA5-Land (already available high-resolution temperature data) and bias-corrected high-resolution temperatures over India at 0.05° is evident (Figures S6D, S7D). Similar to precipitation, we estimated the NSE, R^2^, and RMSE values for the bias-corrected mean monthly maximum and minimum temperatures across India (Tables S2, S3). The application of bias correction showed significant improvements in the skills. Furthermore, we evaluated the NSE, R^2^, and RMSE for the homogenous rainfall zones (Figure S8, Tables S2, S3) and found consistent improvements in the skills after the bias correction. The final bias-corrected high-resolution (0.05°) precipitation and temperature were used to estimate the SPEI drought index over India between 1901–2021.

**Fig. 3:**
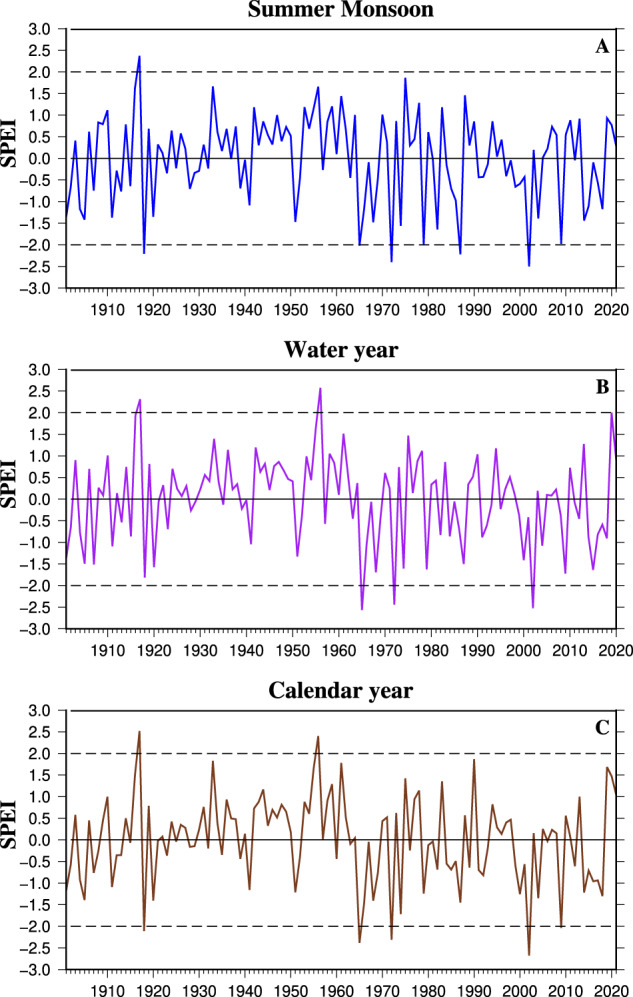
Drought estimates in India based on interannual variability of SPEI. (**A**) Z-score of India’s average 4-month SPEI at the end of September (Summer monsoon: JJAS) for the period 1901–2021, (**B**) Z-score of India’s average 12-month SPEI at the end of May (Water year: June-May) for the period 1901–2020, (**C**) Z-score of India’s average 12-month SPEI at the end of December (Calendar year: January-December) for the period 1901–2021.

To examine if the high-resolution dataset captures the spatial and temporal variability in major droughts, we used the time series of average SPEI over India to assess drought occurrences during the summer monsoon season, water year, and calendar year from 1901 to 2021 (Fig. 3). We calculated the standardized SPEI from the mean SPEI aggregated using the gridded data for an admirative region (state, district, and taluk). The summer monsoon of 2002 ranked as the most severe monsoon season drought followed by 1972, 1987, and 1918, based on SPEI values lower than −2.0 (Fig. 3A). Similarly, the worst events for the water year drought were observed in 1965, 2002, and 1972 (Fig. 3B). The droughts in 2002, 1965, 1972, 1918, and 2009 were identified as the five most exceptional calendar year droughts in India (Fig. 3C). The occurrence of droughts exhibited fluctuations across different decades (Fig. 3). Between 1901 and 1920, there was one extreme/exceptional drought year (SPEI between −3.0 and −1.6). However, from 1921 to 1960, the incidence of drought decreased significantly, with no exceptional drought events recorded during this period. Most of the Indian monsoon region was wet during this period^3^. Subsequently, from 1961 to 1987, the frequency of droughts increased, which was associated with the influence of the El Nino Southern Oscillation^10^. We also estimated the annual drought area coverage (%) between 1901−2021 during the monsoon season, water year, and calendar year in India (Figure S9). We considered the grids with SPEI values below −0.5 to calculate the total drought area. More than 60% of the total geographical area of India was under drought during the exceptional (SPEI less than −2.0) drought events (Figure S9), which signifies the severity of these observed droughts in India.

**Fig. 4:**
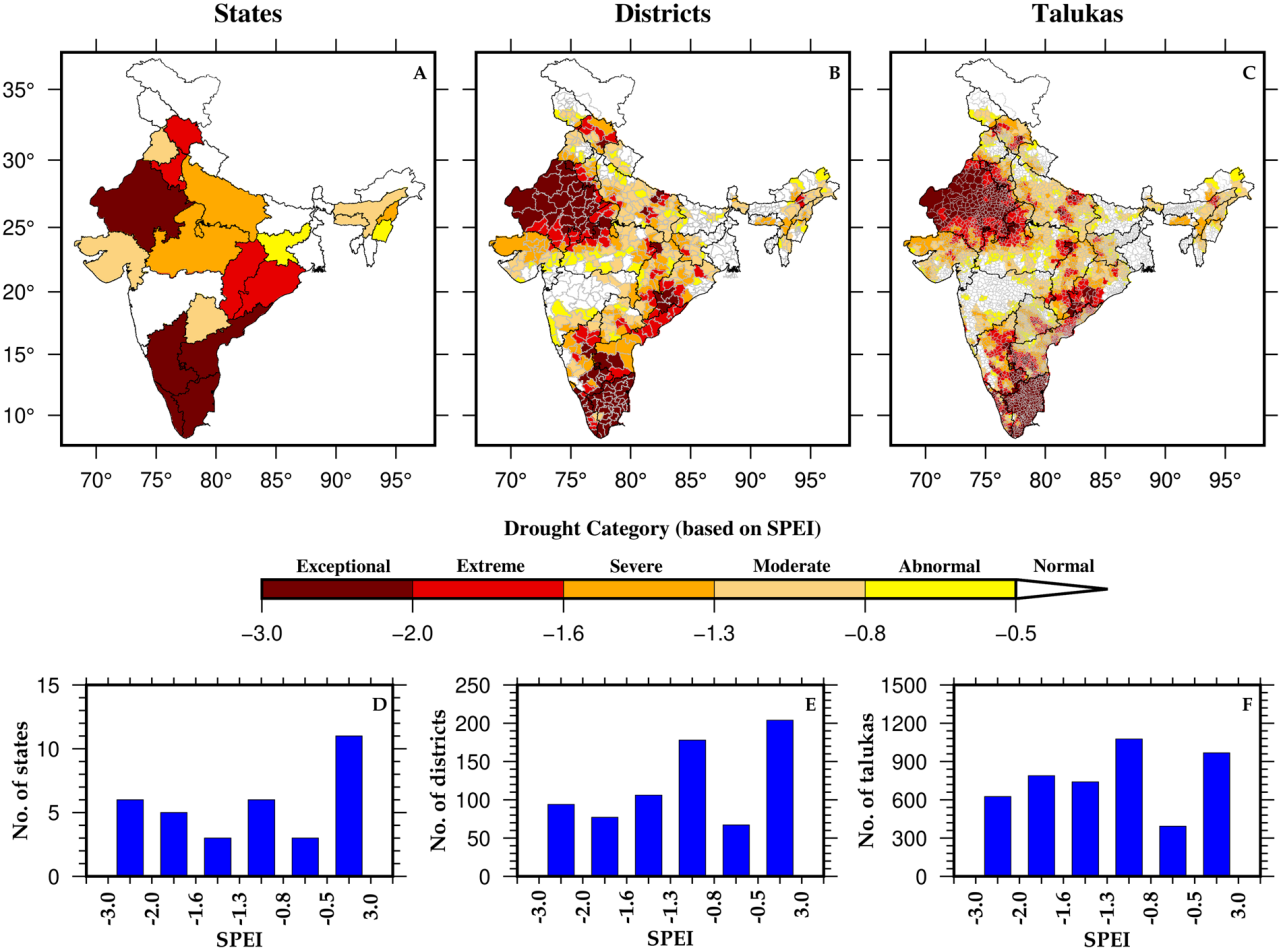
Worst summer monsoon season drought in India (2002) between 1901–2021 based on SPEI. (**A**–**C**) Spatial representation of Z-score of SPEI values across India at State, District, and Taluka (Sub-district) levels. (**D**–**F**) Distribution of States, Districts, and Talukas based on SPEI values for the year 2002.

We examined the drought conditions for states, districts, and talukas during the worst monsoon season (2002), water year (1965), and calendar year (2002) droughts in India (Fig. 4, S10, S11). The peninsular and north-western parts of India were the most affected regions during the 2002 monsoon season drought, whereas the top northernmost part of India remained unaffected (Fig. 4A–C). The drought situation affected more than 23 states, 522 districts, and 3623 talukas, with the SPEI ranging between −2.0 to −0.5 (Fig. 4D–F). Similarly, the central and eastern parts of India were the most affected regions during the worst water year drought in 1965 (Figure S10A–C). More than 80% of the total states (27), districts (584), and talukas (3666) in India were under drought (Figure S10D–F). Moreover, the 2002 calendar year drought significantly affected the eastern, north-western, and southern parts of India (Figure S11A–C). During this period, over 70% of the total states (25), districts (548), and talukas (3676) experienced drought situations (Figure S11D–F). The 1965 water year drought was more severe in terms of areal coverage than the 2002 monsoon season and calendar year droughts (Figure S9).

**Fig. 5:**
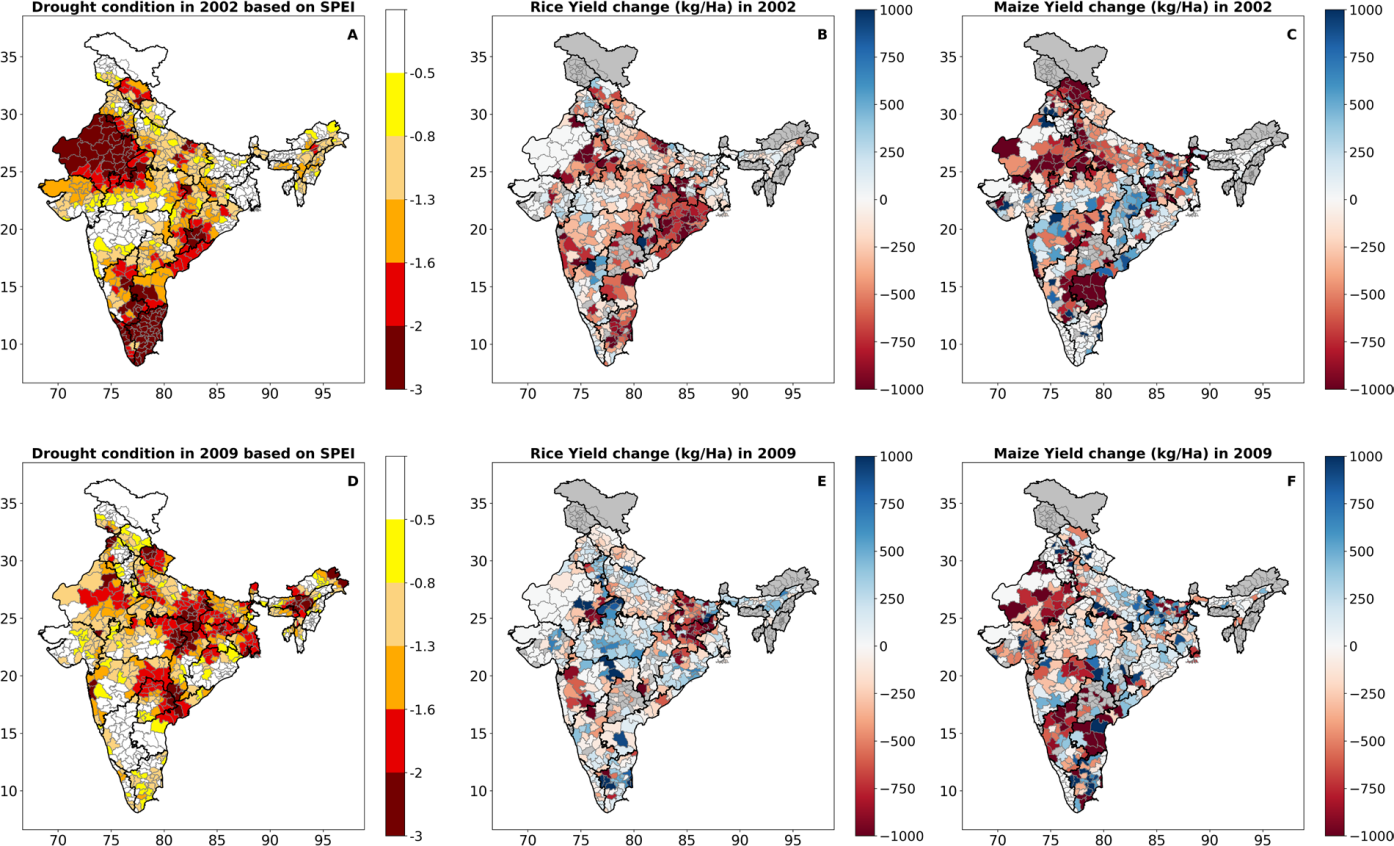
Impact of drought on major crops in India. (**A**) Drought-affected districts in India during the 2002 summer monsoon based on SPEI (Z-score). (**B,C**) Change in the yield (Kilogram/hectare) of Rice and Maize in 2002 compared to 2001 at the district level. (**D**) Drought-affected districts in India during the 2009 summer monsoon based on SPEI (Z-score). (**E,F**) Change in the yield (Kilogram/hectare) of Rice and Maize in 2009 compared to 2008 at the district level. Crop production data was obtained from the ICRISAT database available from the year 1990. The grey colour in the Fig. (**B,C,E,F**) represents missing data. The year 2002 and 2009 were two recent monsoon season droughts (SPEI less than −2.0) in India.

As a next step of data validation, we analyzed the impacts of the summer monsoon season droughts of 2002 and 2009 on the major crop yield in India (Fig. 5). We obtained yearly crop data for Indian districts from the ICRISAT database (http://data.icrisat.org/dld/), available from 1990 onwards and corresponding to India’s district boundaries before 2015. The years 2002 and 2009 witnessed two recent monsoon droughts of exceptional and extreme categories for which crop data is available in the ICRISAT database. The change in yield for a year is calculated by taking the difference between the yield of the current year and the yield of the previous year. We primarily focused on Rice and Maize, which are the two most essential rainy-season crops due to their higher water demands for growth. The impact of the summer monsoon season drought is evident in the production of these two crops (Fig. 5). The 2002 drought mainly affected the north-western, southern, and eastern regions of India, leading to substantial reductions in crop yield in those areas (Fig. 5A–C). On the other hand, the monsoon drought of 2009 had a more pronounced impact on the east-central and north-western regions of India, resulting in a reduction in crop yield in these areas (Fig. 5D–F). While Rice is not a significant crop in north-western India, including Rajasthan and Gujarat (Figure S12A), drought impact on its yield in this region was relatively insignificant. However, the decline in Maize yield in the same region was evident, as north-western states are significant producers of maize in India (Figure S12B). These results emphasize the effectiveness of the high-resolution data in capturing the drought events that cause significant crop loss in drought-affected regions of India.

**Fig. 6:**
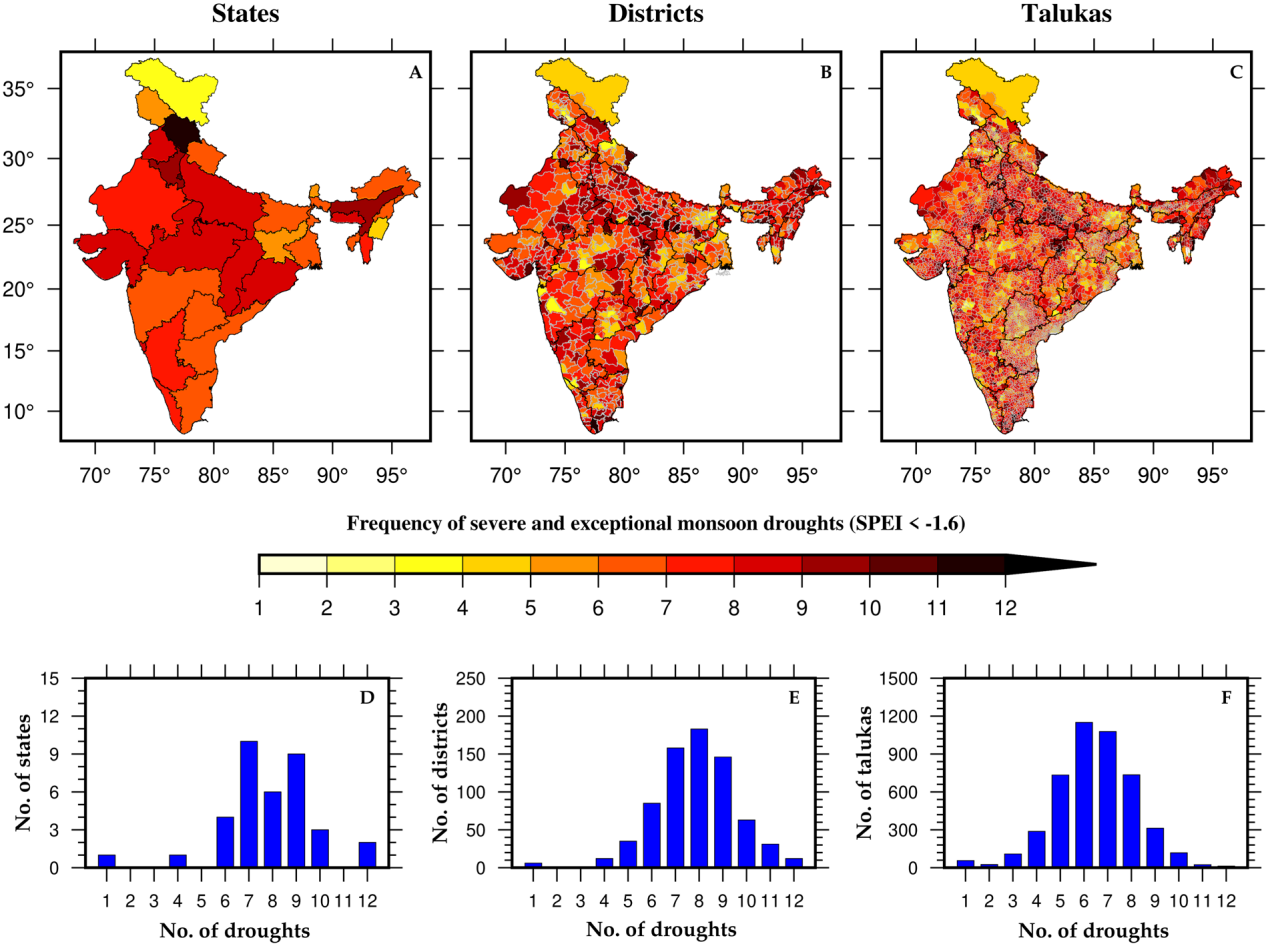
Frequency of severe and exceptional droughts occurred in India. Number of drought events based on Z-score of SPEI values (SPEI less than −1.6) across India between 1901–2021 at (**A**) State, (**B**) District, (**C**) Taluka (Sub-district) levels. (**D**–**F**) Distribution of States, Districts, and Talukas based on the number of droughts events that occurred between 1901–2021.

To further demonstrate the effectiveness of high-resolution data, we analyzed the frequency of severe and exceptional drought events (SPEI less than −1.6) that occurred in India’s states, districts, and talukas between 1901–2021 (Fig. 6). At the state level, the northernmost part of India (Ladakh) has the least frequency of these events, while Himachal Pradesh (just below Ladakh) demonstrated the highest occurrence of such drought events (Fig. 6A). Notably, a high spatial variability was observed within states when examined at the district and taluka levels (Fig. 6B,C). Also, as we move to the higher spatial resolution, the frequency of drought events crossing the threshold (SPEI less than −1.6) increases (Fig. 6A–C). This is because the averaging of SPEI values across larger spatial areas reduces variability, leading to higher z-scores (standardized values). The occurrences of drought events were predominantly clustered between 6 and 10 for the majority of states (Fig. 6D). The number of drought events was concentrated between 6 and 10 for most of the states (Fig. 6D). However, at the district level, the concentration of these events was observed between 5 and 11 occurrences and between 4 and 9 occurrences at the taluka level (Fig. 6E,F).

**Fig. 7:**
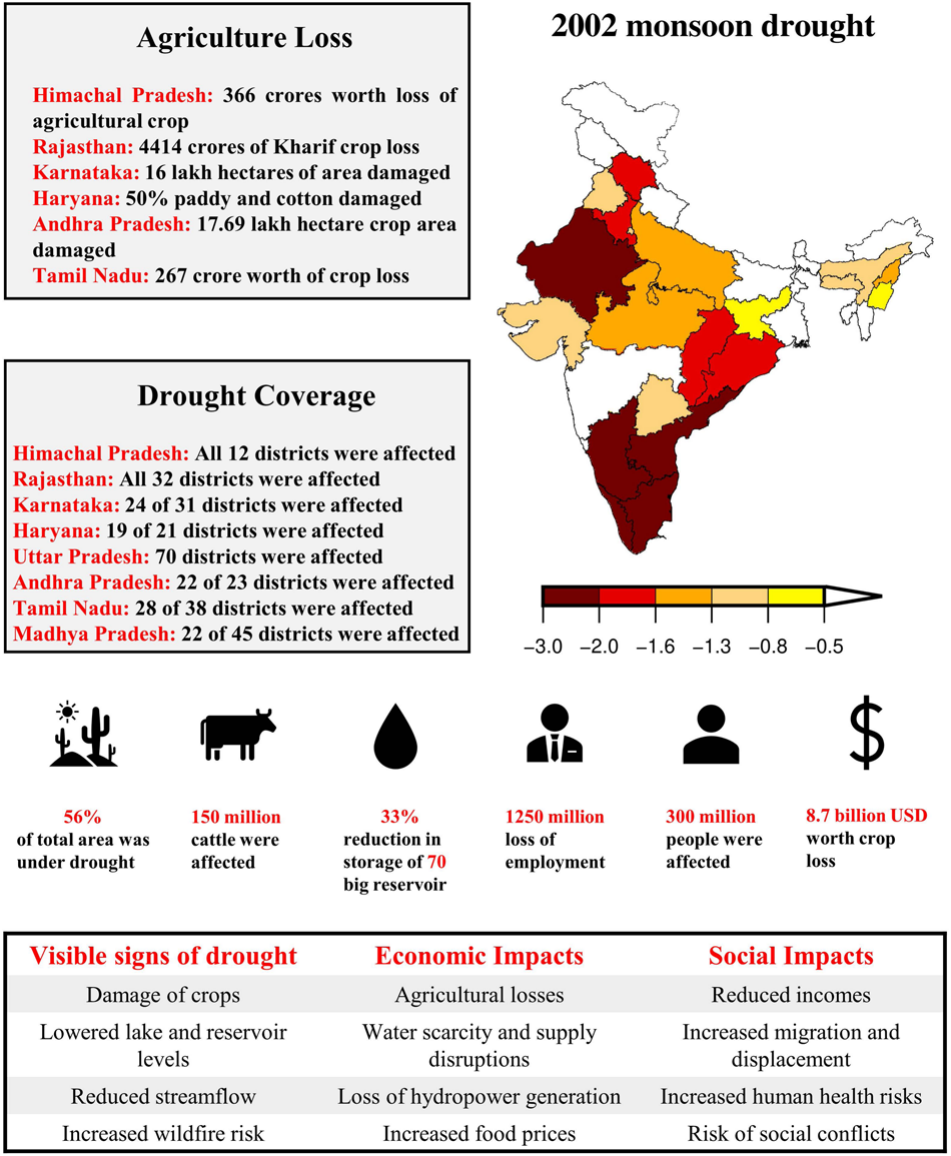
Impacts of the 2002 drought on different sectors of India.

As a next step of our high resolution data validation, we showed^64^ significant impacts of the 2002 drought across various sectors in India (Fig. 7). During this drought, approximately 56% of India’s area experienced moderate to exceptional drought conditions, affecting 300 million people and 150 million cattle (Fig. 7). The economic impact of the drought was also substantial. The country experienced a reduction in per capita income due to the loss of over 1250 million person-days of employment. Additionally, an estimated economic loss of about 8.7 billion USD was reported due to crop damage, which reduced the country’s agricultural gross domestic product (GDP) by 3.1% (Fig. 7).

**Fig. 8:**
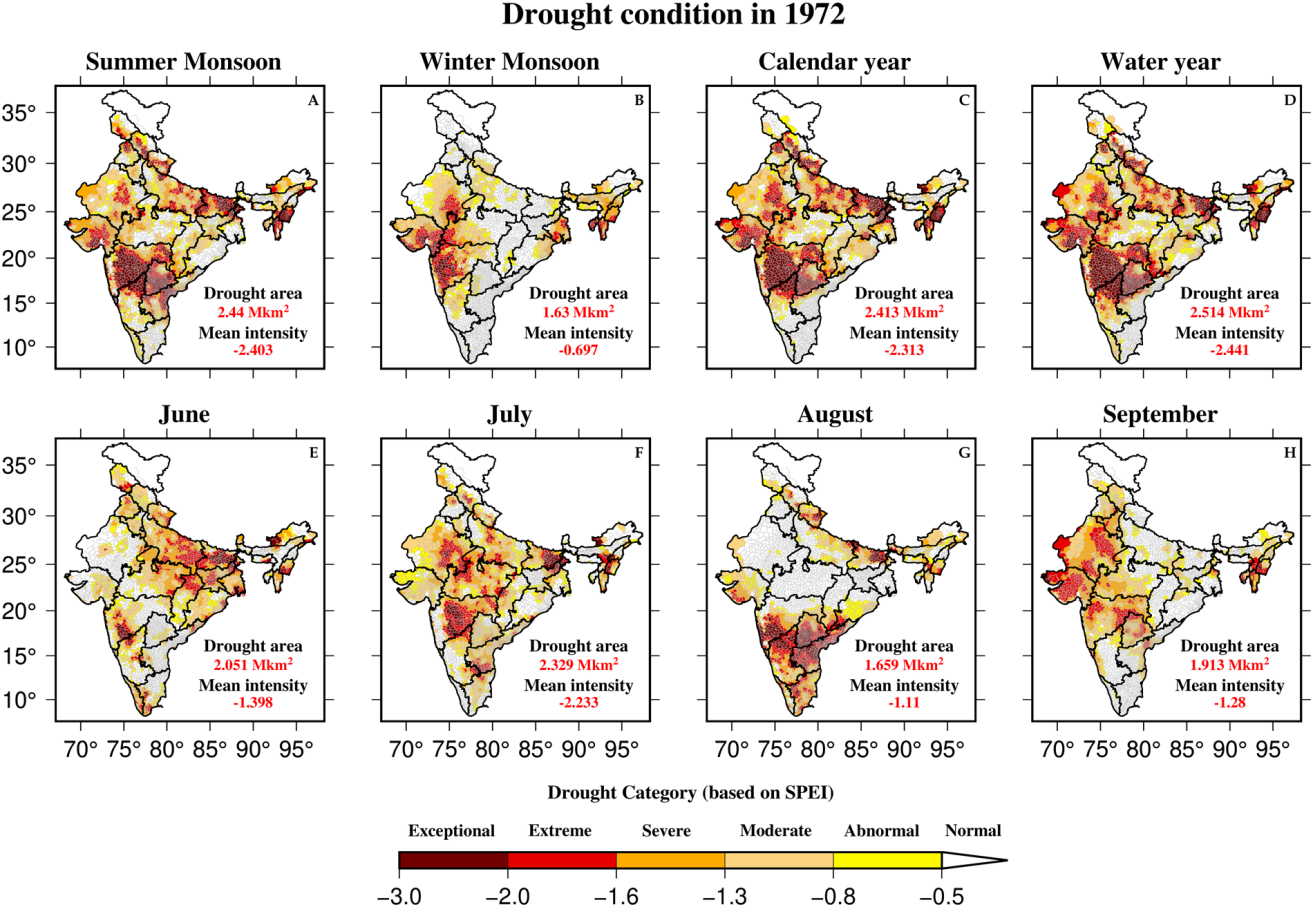
Drought condition in India for different seasons and time scales at taluka level. Drought condition based on SPEI (Z-score) for (**A**) summer monsoon (JJAS), (**B**) winter monsoon (ONDJ), (**C**) calendar year (January-December), (**D**) water year (June-May), (**E**) June, (**F**) July, (**G**) August, (**H**) September are represented at taluka level along with the total drought area in km^2^ (for SPEI less than −0.5) and mean drought intensity.

Finally, using the high-resolution (0.05°) SPEI, we developed the Drought Atlas of India for each year between 1901 and 2020. The atlas includes the taluka-wise drought condition of summer monsoon, winter monsoon, calendar year, water year, and monsoon months (JJAS) for each year. As an example, we show drought condition for 1972 (Fig. 8), which was the second most exceptional monsoon season drought in India (Fig. 3A). The severity of the 1972 drought was exceptionally high for all the selected seasons (except winter monsoon) and for all monsoon months (Fig. 8).

## Usage Notes

The gridded SPEI data are available at 0.05° spatial resolution from 1901 to 2021 at 1-month, 4-month, and 12-month scales. Gridded SPEI data and drought atlas plots can be accessed from the Zenodo repository^62^. Each year’s drought atlas plot shows drought-affected areas of different categories (Normal to Exceptional) across different talukas in India, highlighting the drought-prone areas, which can be directly used for future drought-related studies. High-resolution SPEI data can be used for analyzing the droughts at the basin and sub-basin levels.

We checked the accuracy of bias-corrected data against the reference data and noted significant improvements in its performance. However, despite the bias correction, potential bias may still exist^65,66^. The application of bias correction and interpolation techniques may also introduce random errors in the precipitation and temperature data^42,67^. Moreover, due to limited observations of climate variables, we estimated PET using the Hargreaves method, which may result in an overestimation of PET and drought^4,68,69^.

### Supplementary information

Drought Atlas of India, 1901-2020

## Data Availability

Code to estimate SPEI can be downloaded from: https://github.com/sbegueria/SPEI.
