# Supplementary material for: Drought Atlas of India, 1901–2020
Source: Sci Data. 2024 Jan 2;11:7. doi: 10.1038/s41597-023-02856-y (PMC10762040; doi:10.1038/s41597-023-02856-y)
Supplement: Supplementary file 1 — Drought Atlas of India, 1901-2020 [file 41597_2023_2856_MOESM1_ESM.docx]

**Supplemental Information**

**Drought Atlas of India, 1901-2020**

Dipesh Singh Chuphal^1^, Anuj Prakash Kushwaha^2^, Saran Aadhar^3^ and Vimal Mishra^1, 2^

1 Civil Engineering, Indian Institute of Technology (IIT) Gandhinagar, India

2 Earth Sciences, Indian Institute of Technology (IIT) Gandhinagar, India

3 Civil & Infrastructure Engineering, Indian Institute of Technology (IIT) Jodhpur, India

Corresponding author: Vimal Mishra, [vmishra@iitgn.ac.in](mailto:vmishra@iitgn.ac.in)


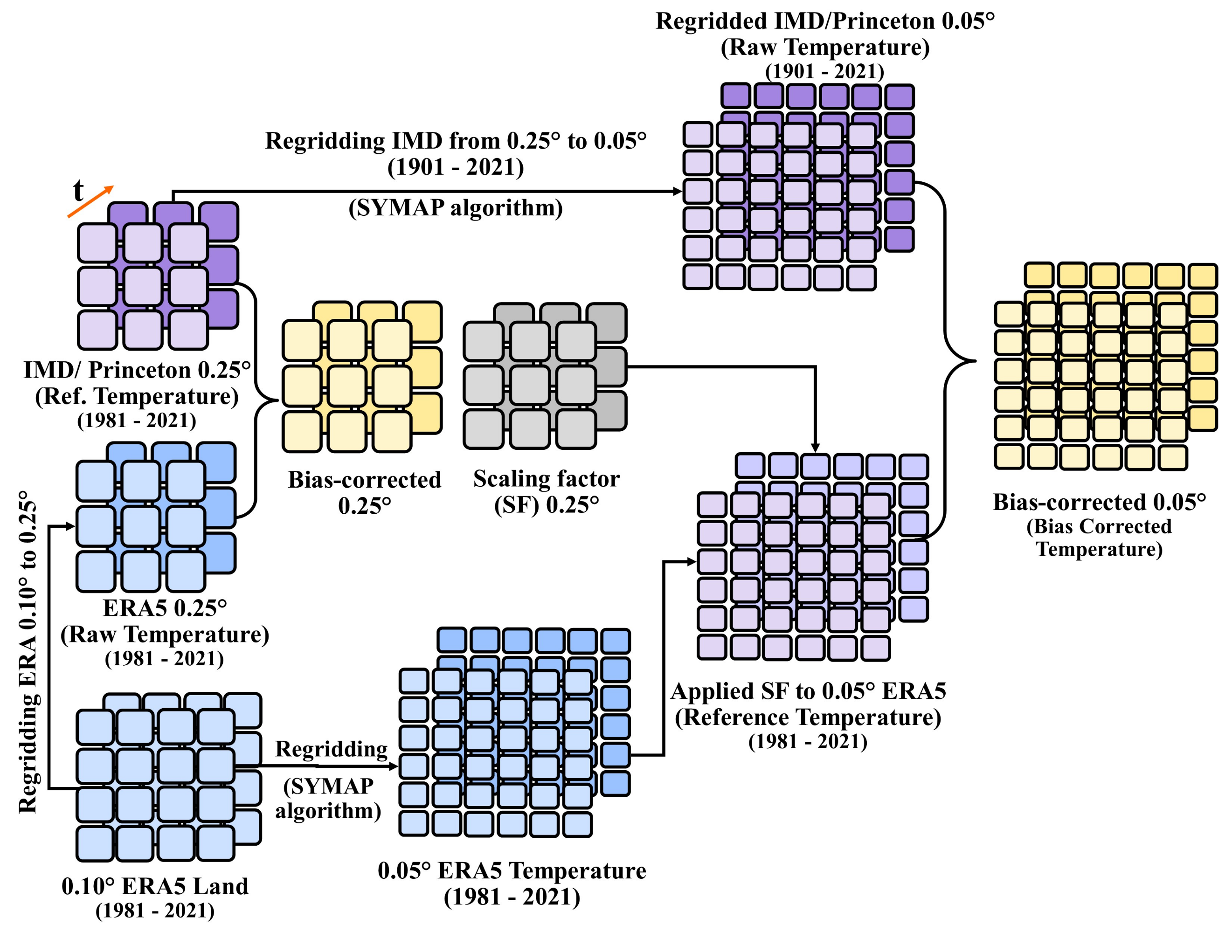


**Figure S1: Steps to construct high-resolution (0.05°) temperature data.**


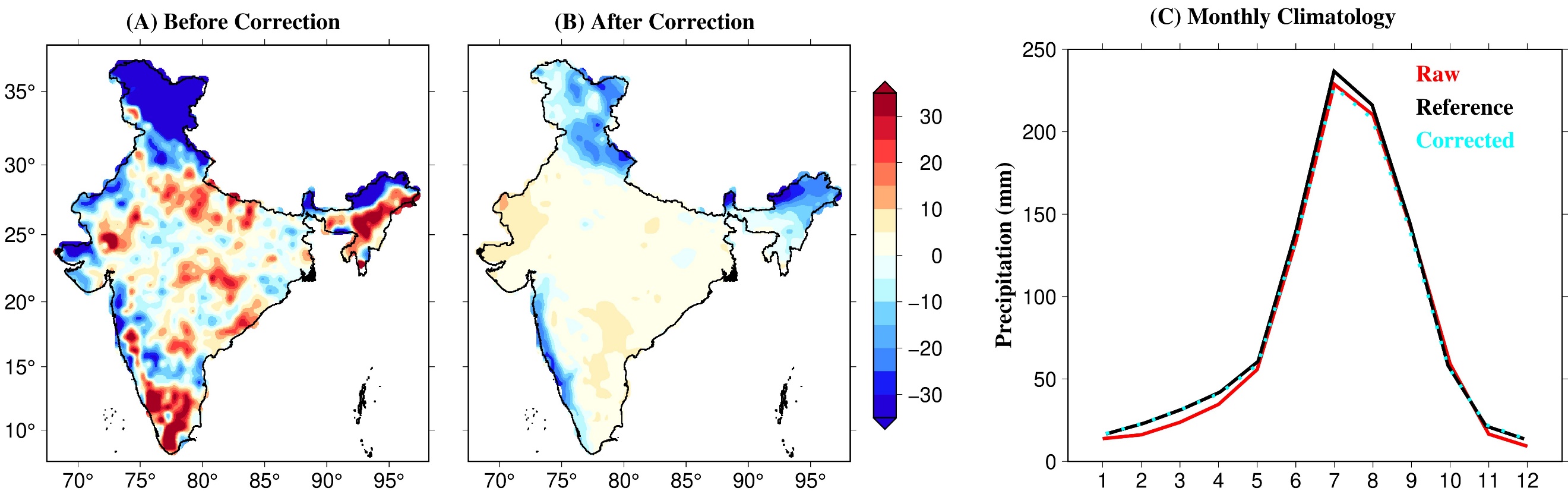


**Figure S2. Bias in CHIRPS precipitation (aggregated to 0.25° from 0.05°) before and after bias correction.**

(A) Percentage (%) bias in raw CHIRPS precipitation aggregated at 0.25° for the period 1981-2016 against reference IMD precipitation. (B) Percentage (%) bias in bias-corrected CHIRPS precipitation at 0.25° against reference IMD precipitation. (C) Mean monthly climatology of raw (CHIRPS), reference (IMD), and bias-corrected (CHIRPS) precipitation at 0.25°.


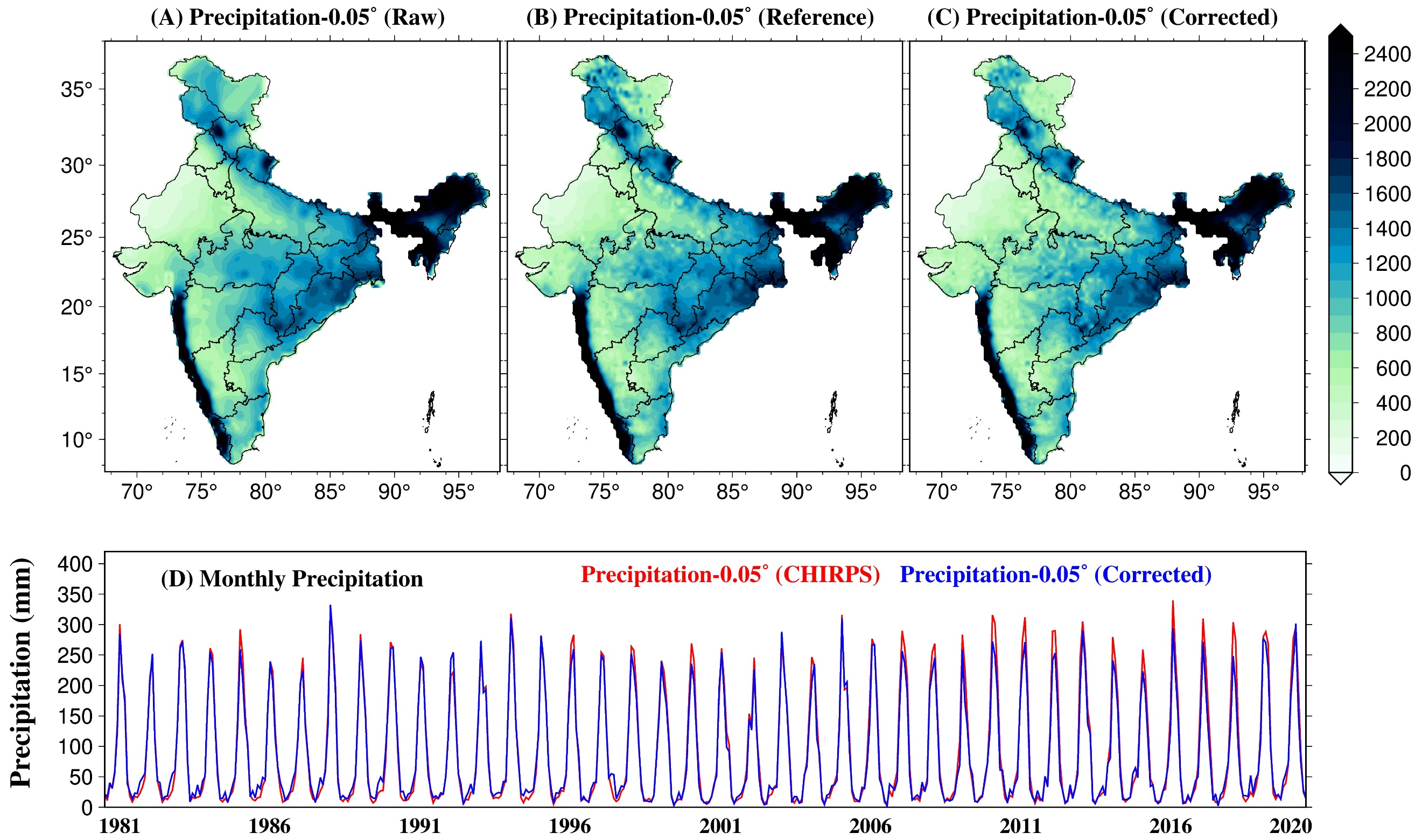


**Figure S3. Long-term mean annual precipitation (mm) over India at 0.05° spatial resolution.**

(A) Mean annual precipitation from IMD (raw) regridded at 0.05° spatial resolution for the period 1981–2021. (B) Mean annual bias-corrected CHIRPS precipitation at 0.05° (reference). (C) Mean annual bias-corrected high-resolution precipitation (corrected). (D) Comparison of mean monthly bias-corrected high-resolution precipitation averaged over India against CHIRPS at 0.05°. The bias correction of CHIRPS precipitation at 0.05° was performed using the scaling factors evaluated during the bias correction of CHIRPS precipitation at 0.25°.


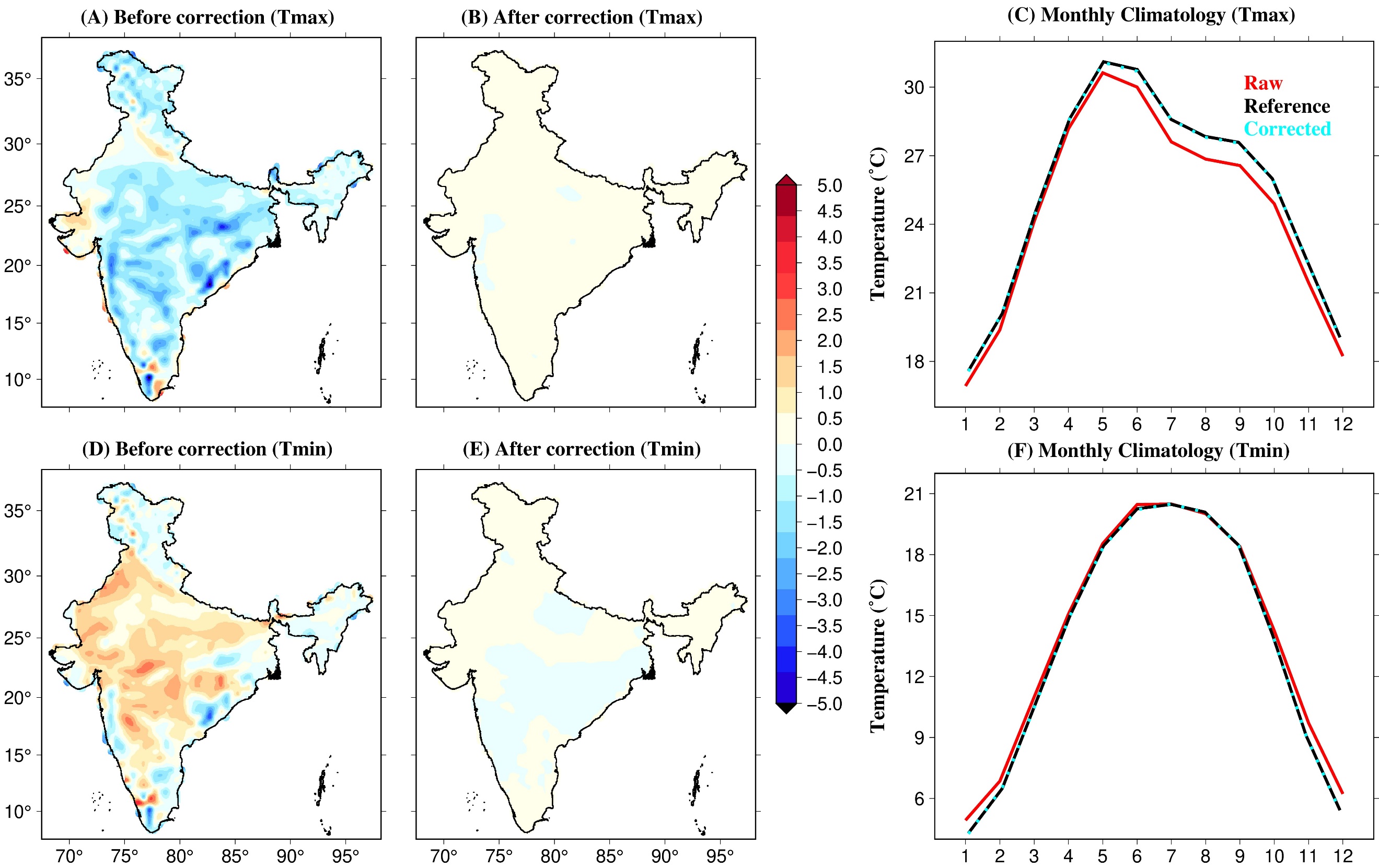


**Figure S4. Bias in ERA5-Land maximum and minimum temperatures (aggregated to 0.25° from 0.1°) before and after correction.**

(A, B) Bias (°C) in mean annual maximum ERA5-Land temperature aggregated at 0.25° before and after bias correction against reference IMD temperature. (D, E) Bias (°C) in mean annual minimum ERA5-Land temperature aggregated at 0.25° before and after bias correction against reference IMD temperature. (C, F) Mean monthly climatology of raw (ERA5-Land), Reference (IMD), and corrected maximum and minimum temperature (Corrected ERA5-Land).


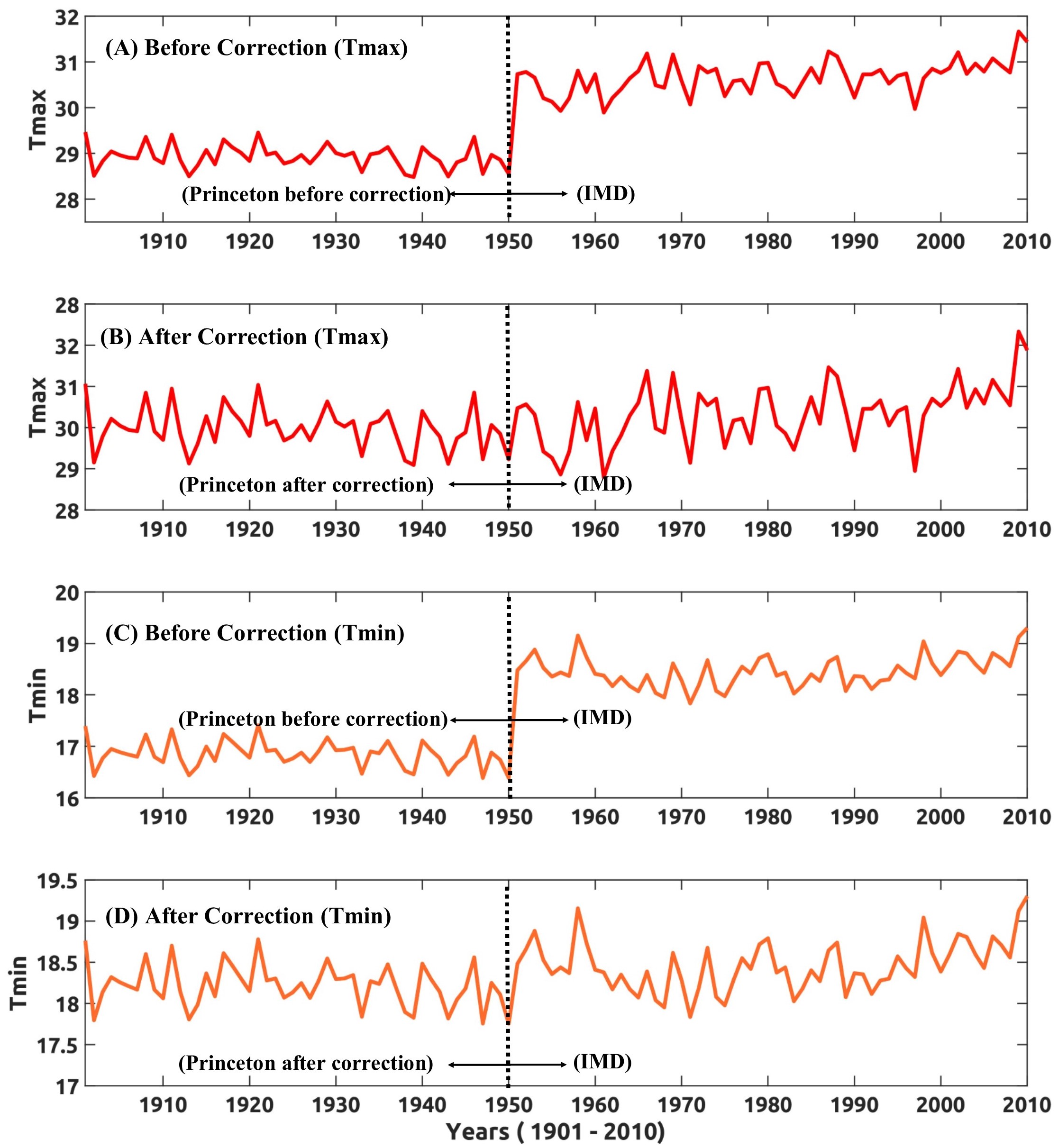


**Figure S5. Bias in maximum and minimum Princeton temperatures before and after bias correction.**

(A, C) Mean annual maximum and minimum temperatures for India from Princeton and IMD temperature. (B, D) Mean annual maximum and minimum temperatures for India from Princeton and IMD temperature after bias correcting Princeton temperature.


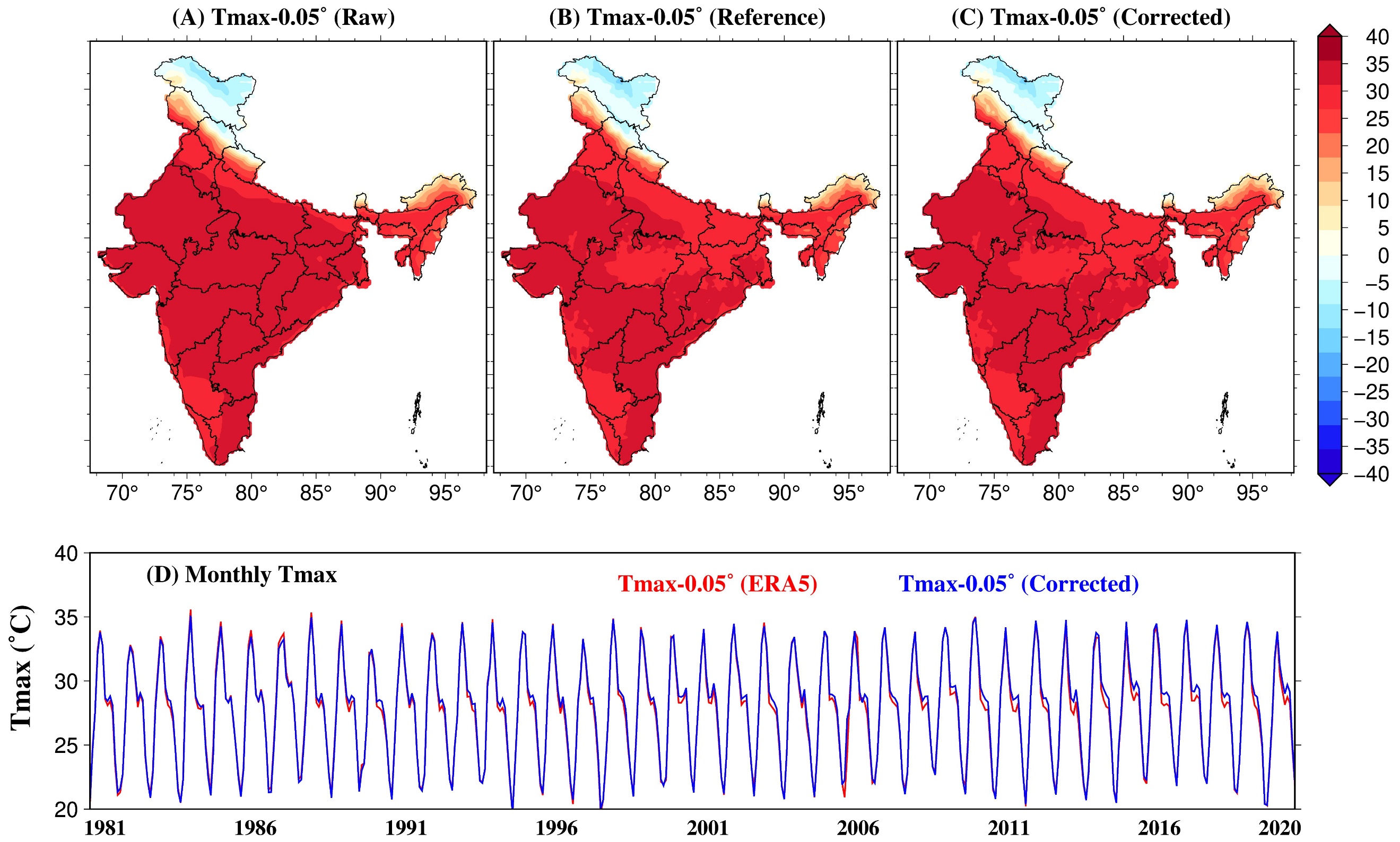


**Figure S6.** **Long-term mean annual maximum temperature (°C) over India at 0.05° spatial resolution.**

(A) Mean annual maximum temperature from IMD (raw) regridded at 0.05° spatial resolution (constructed from ERA5-Land temperature at 0.1° using elevation-based SYMAP algorithm) for the period 1981–2021. (B) mean annual bias-corrected maximum ERA5-Land temperature at 0.05° (reference). (C) Bias corrected mean annual high-resolution maximum temperature (corrected). (D) Comparison of mean monthly bias-corrected high-resolution maximum temperature averaged over India against ERA5-Land at 0.05°. The bias correction of ERA5-Land temperature at 0.05° was performed using the scaling factors evaluated during the bias correction of ERA5-Land temperature at 0.25°.


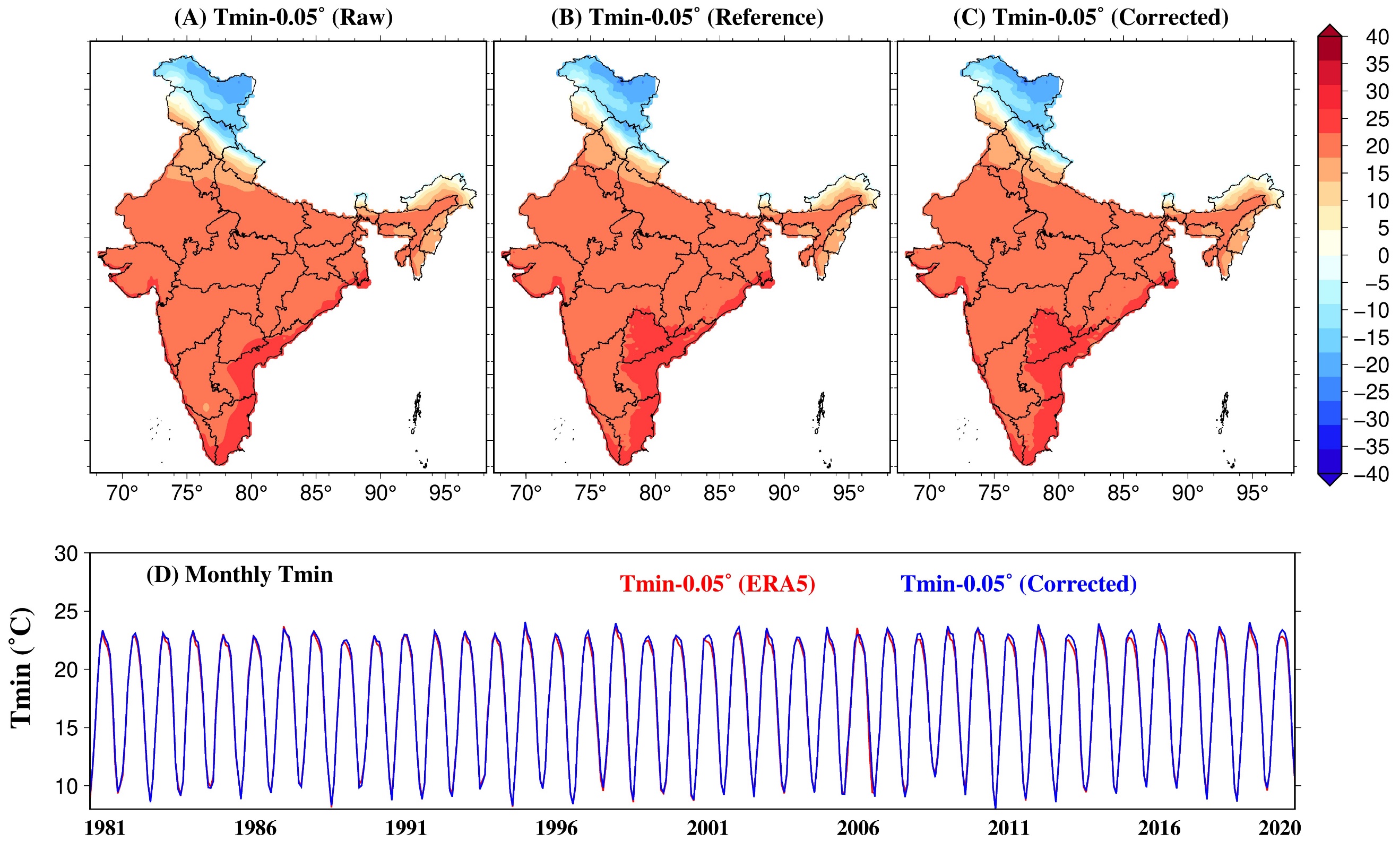


**Figure S7.** **Long-term mean annual minimum temperature (°C) over India at 0.05° spatial resolution.**

(A) Mean annual minimum temperature from IMD (raw) regridded at 0.05° spatial resolution (constructed from ERA5-Land temperature at 0.1° using elevation-based SYMAP algorithm) for the period 1981–2021. (B) Mean annual bias-corrected minimum ERA5-Land temperature at 0.05° (reference). (C) Bias corrected mean annual high-resolution minimum temperature (corrected). (D) Comparison of mean monthly bias-corrected high-resolution minimum temperature averaged over India against ERA5-Land at 0.05°. The bias correction of ERA5-Land temperature at 0.05° was performed using the scaling factors evaluated during the bias correction of ERA5-Land temperature at 0.25°.


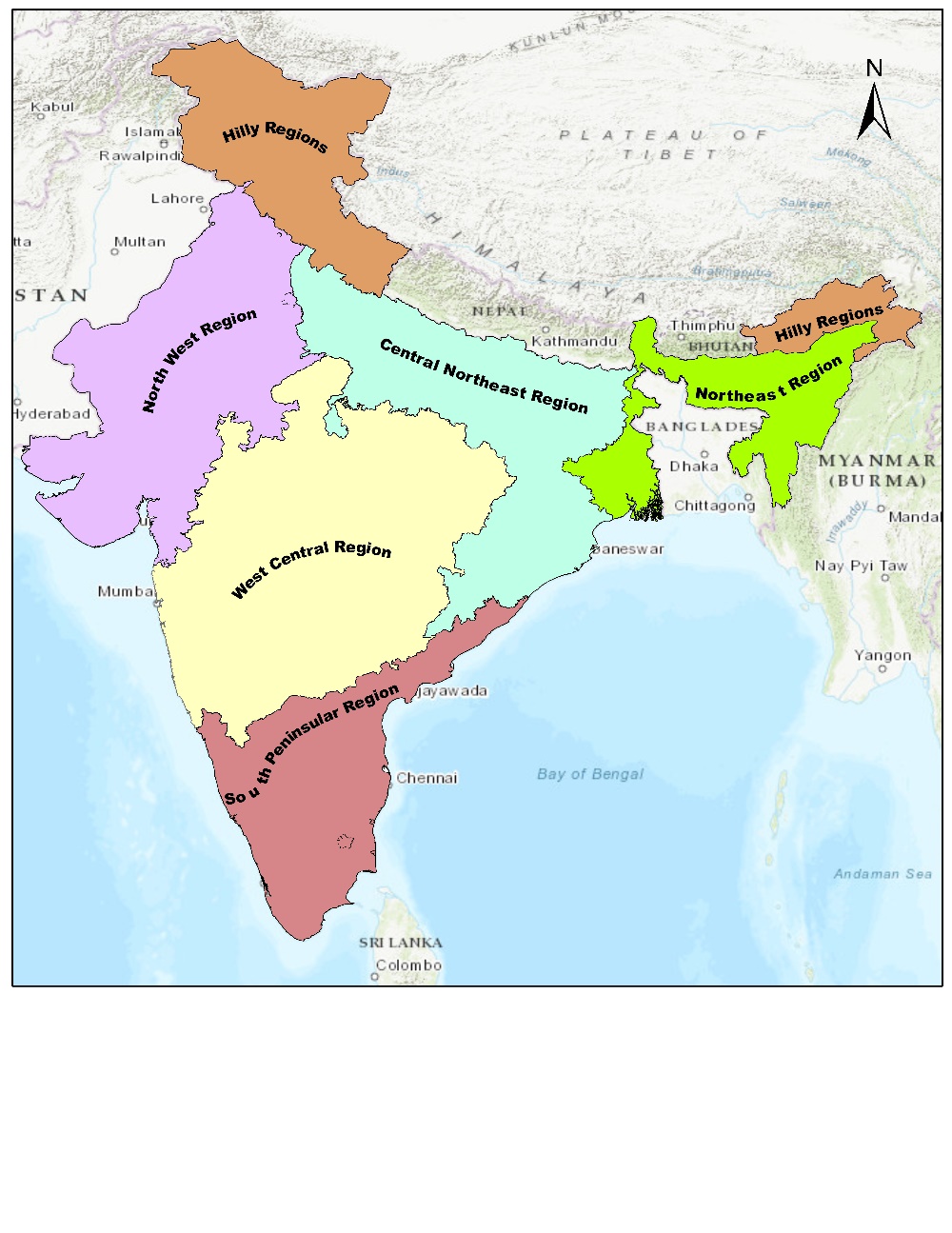


**Figure S8. Homogeneous rainfall zones in India.**


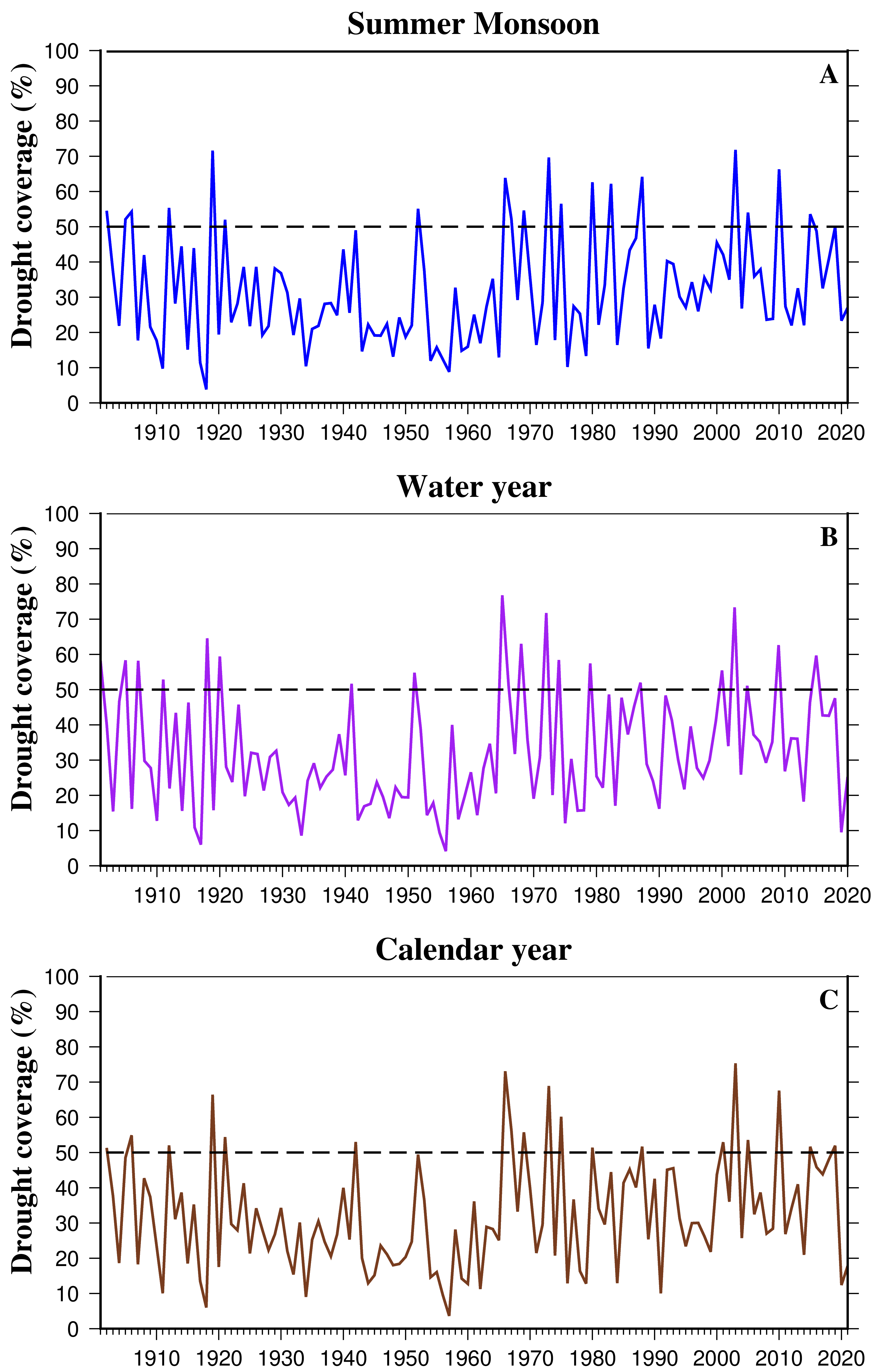


**Figure S9: Drought coverage (%) in India based on interannual variability of SPEI.**

Drought area estimation considers grids with SPEI values (Z-score) below -0.5. Total geographical area (%) of India under drought for (A) Summer Monsoon for the period 1901-2021, (B) Water year for the period 1901-2020, (C) Calendar year for the period 1901-2021.


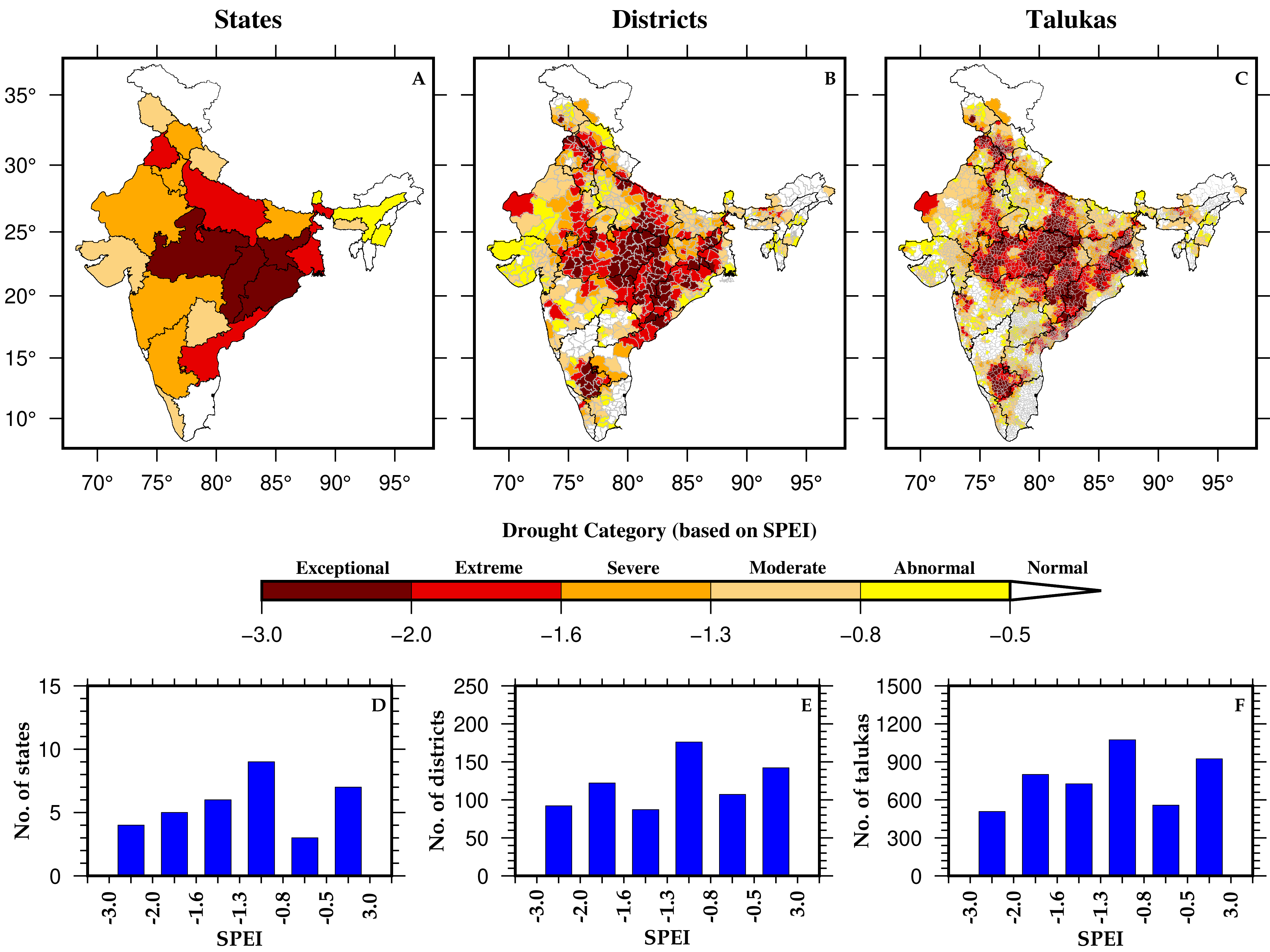


**Figure S10: Worst water year drought in India (1965) between 1901-2020 based on SPEI.**

(A, B, C) Spatial representation of Z-score of SPEI values across India at State, District, and Taluka (Sub-district) levels. (D, E, F) Distribution of States, Districts, and Talukas based on SPEI values.


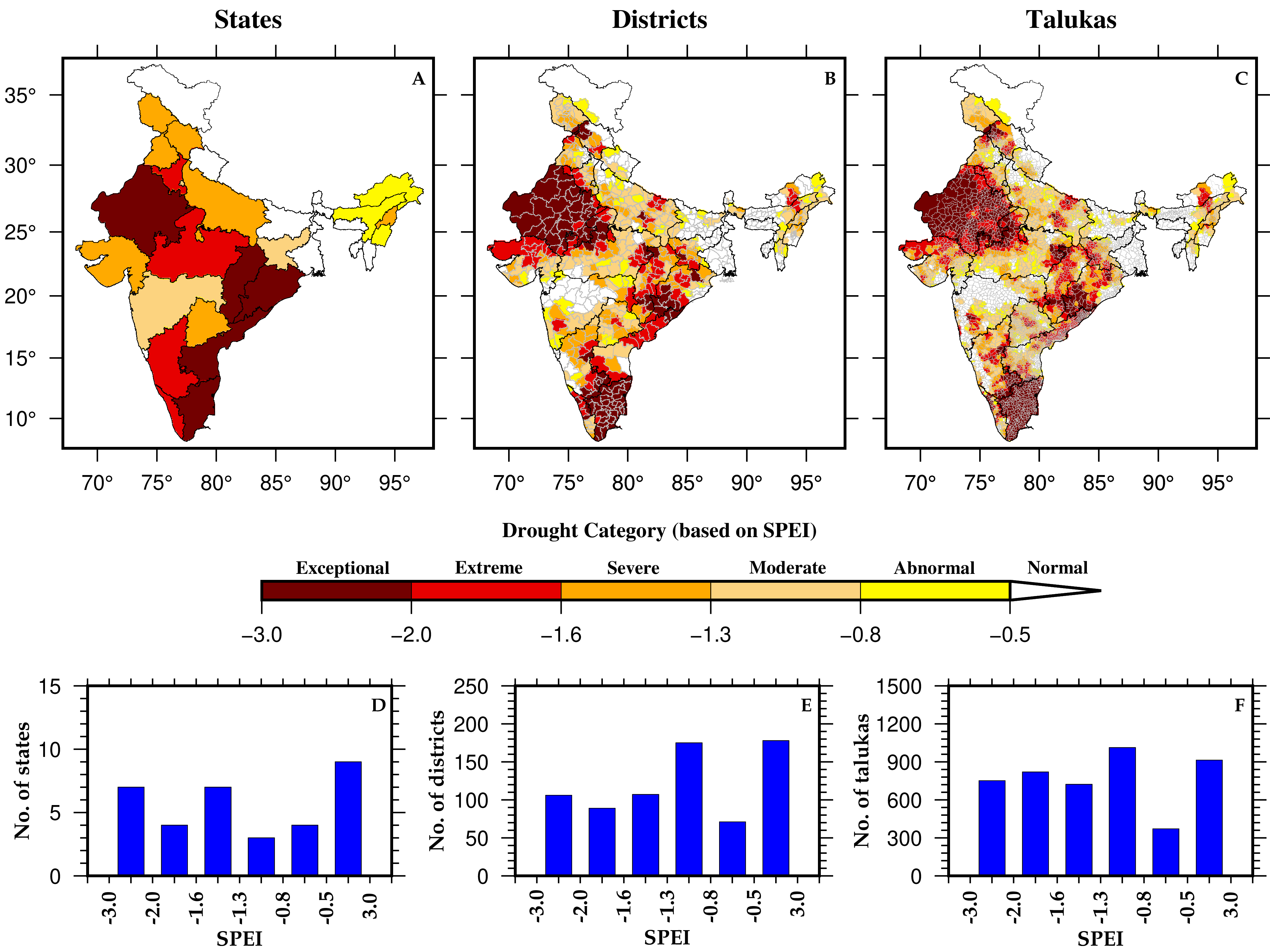


**Figure S11: Worst calendar year drought in India (2002) between 1901-2021 based on SPEI.**

(A, B, C) Spatial representation of Z-score of SPEI values across India at State, District, and Taluka (Sub-district) levels. (D, E, F) Distribution of States, Districts, and Talukas based on SPEI values.


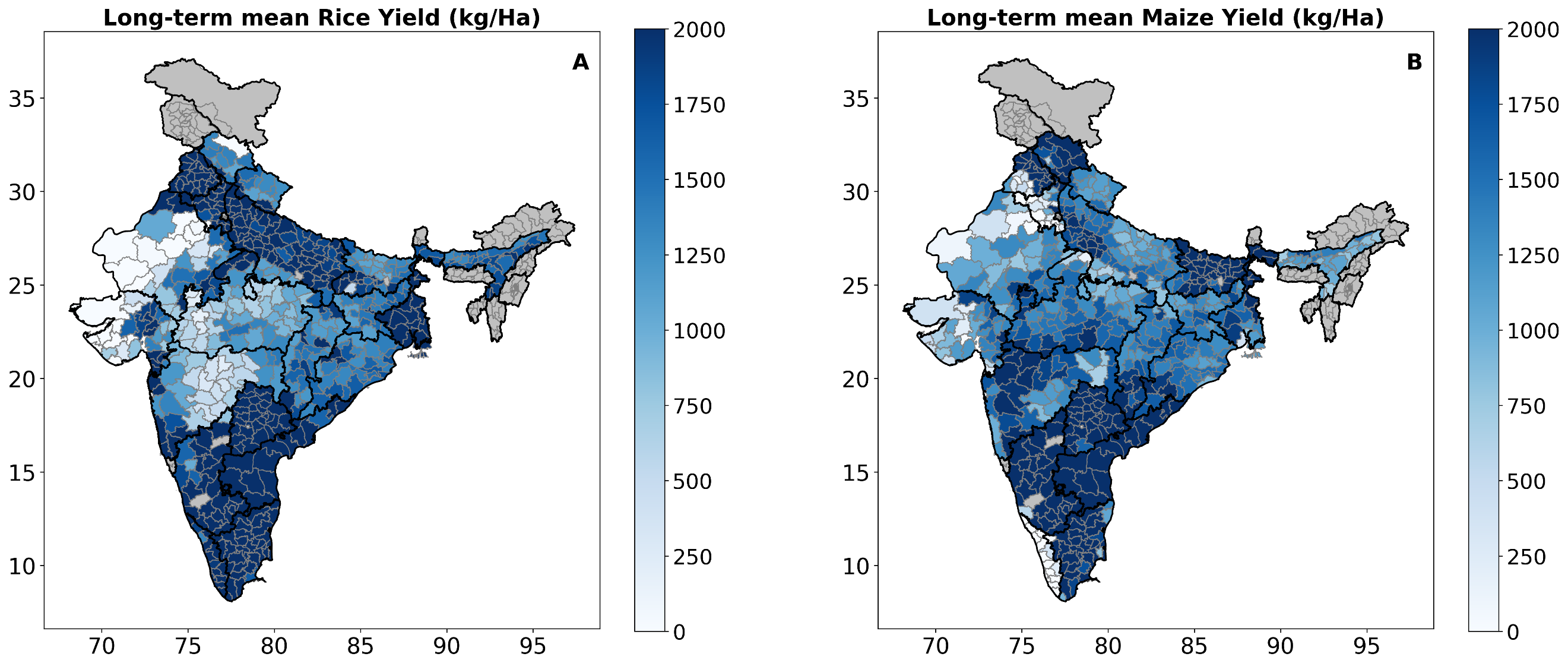


**Figure S12: Long-term mean (A) rice yield (kg/Ha) and (B) maize yield (kg/Ha) in different districts of India.**

**Table S1. Comparison of performance of the bias correction in precipitation.**

| Region | NSE | | R2 | | RMSE | |
| --- | --- | --- | --- | --- | --- | --- |
|  | Before | After | Before | After | Before | After |
| India (Whole) | 0.979 | 0.983 | 0.985 | 0.986 | 11.11 | 8.22 |
| Central Northeast | 0.968 | 0.970 | 0.972 | 0.971 | 20.38 | 19.52 |
| Hilly Regions | 0.360 | 0.785 | 0.739 | 0.797 | 57.52 | 33.32 |
| Northeast | 0.954 | 0.970 | 0.959 | 0.970 | 34.10 | 27.68 |
| Northwest | 0.961 | 0.969 | 0.963 | 0.969 | 13.41 | 12.64 |
| South Peninsular | 0.863 | 0.927 | 0.909 | 0.928 | 27.70 | 20.18 |
| West Central | 0.977 | 0.973 | 0.977 | 0.976 | 17.34 | 11.12 |

**Table S2. Comparison of performance of the bias correction in maximum temperature.**

| Region | NSE | | R2 | | RMSE | |
| --- | --- | --- | --- | --- | --- | --- |
|  | Before | After | Before | After | Before | After |
| India (Whole) | 0.964 | 0.992 | 0.993 | 0.995 | 0.85 | 0.33 |
| Central Northeast | 0.929 | 0.990 | 0.976 | 0.990 | 1.20 | 0.46 |
| Hilly Regions | 0.988 | 0.997 | 0.997 | 0.997 | 0.81 | 0.39 |
| Northeast | 0.950 | 0.989 | 0.983 | 0.990 | 0.66 | 0.31 |
| Northwest | 0.982 | 0.989 | 0.990 | 0.990 | 0.65 | 0.50 |
| South Peninsular | 0.873 | 0.975 | 0.970 | 0.977 | 0.82 | 0.36 |
| West Central | 0.878 | 0.989 | 0.983 | 0.989 | 1.35 | 0.40 |

**Table S3. Comparison of performance of the bias correction in minimum temperature.**

| Region | NSE | | R2 | | RMSE | |
| --- | --- | --- | --- | --- | --- | --- |
|  | Before | After | Before | After | Before | After |
| India (Whole) | 0.994 | 0.998 | 0.997 | 0.998 | 0.47 | 0.24 |
| Central Northeast | 0.983 | 0.996 | 0.992 | 0.996 | 0.74 | 0.36 |
| Hilly Regions | 0.996 | 0.998 | 0.996 | 0.998 | 0.49 | 0.36 |
| Northeast | 0.996 | 0.998 | 0.997 | 0.998 | 0.43 | 0.31 |
| Northwest | 0.968 | 0.996 | 0.993 | 0.996 | 1.13 | 0.42 |
| South Peninsular | 0.984 | 0.997 | 0.996 | 0.997 | 0.51 | 0.24 |
| West Central | 0.948 | 0.995 | 0.987 | 0.995 | 1.03 | 0.33 |
